# Italian Emergency Department Visits and Hospitalizations for Outpatients’ Adverse Drug Events: 12-Year Active Pharmacovigilance Surveillance (The MEREAFaPS Study)

**DOI:** 10.3389/fphar.2020.00412

**Published:** 2020-04-06

**Authors:** Niccolò Lombardi, Giada Crescioli, Alessandra Bettiol, Marco Tuccori, Annalisa Capuano, Roberto Bonaiuti, Alessandro Mugelli, Mauro Venegoni, Giuseppe Danilo Vighi, Alfredo Vannacci, the MEREAFaPS Study group, Maria Luisa Aiezza

**Affiliations:** ^1^Section of Pharmacology and Toxicology, Department of Neurosciences, Psychology, Drug Research and Child Health, University of Florence, Florence, Italy; ^2^Tuscan Regional Centre of Pharmacovigilance, Florence, Italy; ^3^Unit of Adverse Drug Reactions Monitoring, Department of Clinical and Experimental Medicine, University of Pisa, Pisa, Italy; ^4^Section of Pharmacology “L. Donatelli”, Department of Experimental Medicine, Campania Regional Centre for Pharmacovigilance and Pharmacoepidemiology, University of Campania “Luigi Vanvitelli”, Naples, Italy; ^5^Pharmacology Unit, Department of Diagnostics and Public Health, University of Verona, Verona, Italy; ^6^Internal Medicine, Medical Department, Vimercate Hospital, ASST di Vimercate, Vimercate, Italy

**Keywords:** emergency department, hospitalization, adverse drug events, pharmacovigilance, drug safety, preventability, seriousness

## Abstract

**Background:**

Adverse drug event (ADEs) are a significant cause of emergency department (ED) visits and consequent hospitalization. Preventing ADEs and their related ED visits in outpatients remains a public health safety challenge. In this context, the aims of the present study were to describe the frequency, seriousness and preventability of outpatients’ ADE-related ED visits and hospitalizations in the Italian general population, and to identify the presence of potential predictors of ADE-related hospitalization.

**Methods:**

We performed a nationwide, multicentre, observational, retrospective study based on reports of suspected ADEs collected between January 1, 2007 and December 31, 2018 in 94 EDs involved in the MEREAFaPS project. Patients’ demographic characteristics, their clinical status, suspected and concomitant drugs, ADE description, and its degree of seriousness, were collected. Causality and preventability were assessed using validated algorithms, and logistic regression analyses were used to estimate the reporting odds ratios (RORs) with 95% confidence intervals (CIs) of ADE-related hospitalization, considering the following covariates: age, sex, ethnicity, number of implicated medications, parenteral administration, presence of interaction, therapeutic error, and/or complementary and alternative medicines (CAM).

**Results:**

Within 12 years, 61,855 reports of suspected ADE were collected, of which 18,918 (30.6%) resulted in hospitalization (ADE defined as serious). Patients were mostly female (56.6%) and Caucasians (87.7%), with a mean age of 57.5 ± 25.0 years. 58% of patients were treated with more than two drugs, and 47% of ADEs leading to hospitalization were preventable. Anticoagulants, antibiotics, and nonsteroidal anti-inflammatory drugs (NSAIDs) were the most frequently implicated agents for ED visits and/or hospitalization, which included clinically significant ADEs, such as haemorrhage for anticoagulants, moderate to severe allergic reactions for antibiotics, and dermatologic reactions and gastrointestinal disturbances for NSAIDs. Older age (1.54 [1.48–1.60]), higher number of concomitantly taken drugs (2.22 [2.14–2.31]), the presence of drug-drug interactions (1.52 [1.28–1.81]), and therapeutic error (1.54 [1.34–1.78]), were significantly associated with an increased risk of hospitalization.

**Conclusion:**

Our long-term active pharmacovigilance study in ED provided a valid estimation of ADE-related hospitalization in a representative sample of the Italian general population and can suggest further focus on medication safety in outpatients, in order to early recognise and prevent ADEs.

## Introduction

Adverse drug events (ADEs) are the most common cause of iatrogenic harm in health care and have received attention in national patient safety initiatives (Shehab et al., 2016). Moreover, ADEs are a significant cause of emergency department (ED) visits and hospitalizations (Budnitz et al., 2011).

In outpatients, in which 90% of prescription drug expenditures occur (Shehab et al., 2016), an early identification and prevention of ADEs remain a public health and patient safety challenge worldwide, with efforts often focused on medication abuse/misuse, medication errors, and reducing potentially inappropriate prescribing for general population, particularly in children (Carnovale et al., 2014; Lombardi et al., 2018) and elderly, as defined by the Beers criteria (By the American Geriatrics Society Beers Criteria Update Expert, 2015). Patients in primary care and some post-acute care settings can have complex medication regimens, at times prescribed by multiple clinicians, with far less monitoring compared with hospitalized patients.

EDs are an essential part of health care systems, serving as an interface between hospitals and communities, and could constitute the most important source of information about the clinical and economic characteristics of ADEs (Zed et al., 2013; Carnovale et al., 2014).

Several studies have been published on ADEs as a cause of ED admissions, but none of those available in the literature has been conducted at national level and for a long observational period (Perrone et al., 2014; Lombardi et al., 2018; Lombardi et al., 2019). Furthermore, few studies evaluated the presence of specific variables among population and/or medication characteristics which can contribute to an ADE as a cause of hospitalization.

In this context, assuming the presence of predictors of hospitalization related to ADEs in the general population, we first performed this study to describe the frequency, preventability and seriousness of ADEs in Italy and, subsequently, for estimate the risk of hospitalization associated to ADEs, by means of a 12-year active pharmacovigilance surveillance in EDs. For the first time the characteristics of the MEREAFaPS Study database have been described.

## Material and Methods

This is an observational retrospective study performed on data retrieved by pharmacovigilance reports of suspected ADE collected between January 1, 2007 and December 31, 2018 in the EDs participating to the MEREAFaPS Study *(“Monitoraggio Epidemiologico delle Reazioni e degli Eventi Avversi da Farmaci in Pronto Soccorso” - “Epidemiological Monitoring of Adverse Drug Reactions and Events leading to Emergency Department”*), an on-going multicentre study of active pharmacovigilance (Lombardi et al., 2018; Lombardi et al., 2019). This is the first nationwide pharmacovigilance study performed in Italy with an “active” approach and based on electronic ED medical records containing detailed information on patient populations, thereby allowing consideration of modifying factors such as polypharmacy and comorbidity, as well as sociodemographic characteristics.

The study involves a total of 94 EDs belonging to general hospitals serving different areas of Italy. These hospitals are equally distributed through the national territory, in five Italian Regions: Lombardy and Piedmont (north), Tuscany and Emilia-Romagna (centre), and Campania (south). The EDs involved in the present study allow to reach an estimated coverage of over 45% of the Italian population (more than 28millions of inhabitants) (Italian Statistics Bureau (ISTAT), 2018).

All ADEs leading to ED visit were collected from the ED clinical charts and hospitalization data were collected from the hospitals discharge database. ED trained monitors (i) evaluated all ED visits, consulting ED clinical charts and hospital discharge database, (ii) identified those related to ADEs and, following the Italian pharmacovigilance legislation (Mazzitello et al., 2013), (iii) filled out the specific report form. ED trained monitors performed all the aforementioned actions (i, ii, iii) evaluating both electronic and paper-based medical records. An *ad hoc* MEREAFaPS database was constructed, retrieving the following information: (1) patients’ demographic characteristics (age, gender, ethnic group); (2) patients’ clinical status on ED admission; (3) suspected medications (for each one, administration route, therapy duration, dosages, and therapeutic indication were recorded); (4) use of complementary and alternative medicines (CAM); (5) ADEs description; (6) ADEs outcome (resolution with sequelae, still unresolved, complete resolution, improvement, death, and not available).

Suspected and concomitant medications were classified according to the Anatomical Therapeutic Chemical (ATC) classification system. ADEs reported from outpatients having at least one clinical manifestation related to any medication were included in the analysis, considering all ATC classes. Patients who developed an ADE while in the ED were excluded. ADEs description according to diagnosis and symptoms was coded using the Medical Dictionary for Regulatory Activities (MedDRA) and organized by System Organ Class (SOC) and Preferred Term (PT) (Lombardi et al., 2018; Lombardi et al., 2019).

A multidisciplinary team composed by experts in clinical pharmacology (NL, GC, AC, AM, GDV), toxicology (AV, MV), and pharmcoepidemiology (AB, MT, RB), performed a clinical evaluation of cases included in the analysis, in order to assess the causality relationship between the suspected medications and their related ADEs (Naranjo et al., 1981). In particular, the sections of the Naranjo scale concerning to dechallenge and/or rechallenge were taken into consideration. In pharmacovigilance, “dechallenge” refers to the stopping of the suspected drug, usually after an ADE or at the end of a planned treatment. Dechallenges may be complete or partial. That is, the drug is fully stopped or decreased in dose and the ADE may fully disappear or only partially decrease. A positive dechallenge refers to the ADE disappearing after the stopping of the drug. On the contrary, a negative dechallenge refers to the ADE not disappearing after the stopping of the drug. Moreover, “rechallenge” refers to the restarting of the same suspected drug after having stopped it, usually for an ADE. Rechallenges may also be complete or partial. A positive rechallenge refers to the ADE recurring after restarting the drug. To have this occur, the ADE had to have previously disappeared after the dechallenge in order for it to restart. A negative rechallenge is the case where the ADE does not recur after the drug is restarted. It is important to consider that the applicability of these two concepts can be affected by certain limitation (Mittal and Gupta, 2015). In particular, dechallenge may not be applicable where the suspected drug is a one-dose treatment (i.e., vaccine), ADE resulting in death or occurring after discontinuation of drug. Moreover, the evaluation can be difficult to apply, such as in irreversible or long-lasting reaction. Finally, dechallenge cannot be addressed in cases of ADEs showing spontaneous recovery despite continuation of therapy. Considering rechallenge, it may range from a similar episode in the patient’s anamnesis to a true planned prospective re-exposure. Because of ethical and clinical concerns, a true rechallenge is a generally very rare evenience, particularly in serious ADEs. All of these limitations will be considered when applying the Naranjo scale.

Preventability of ADEs (categorized as definitely or probably preventable, or not preventable) was assessed using the Schumock and Thornton algorithm (Schumock and Thornton, 1992). Considering the specific section of the pharmacovigilance report form (available from June 2012) and according to Good Pharmacovigilance Practices (GVP) - Module VI (Rev 2), monitors retrieved information concerning the cause of ADEs, and recorded, only when reported, whether ADEs were due to misuse/abuse/medication error/overdose or drug-drug interactions (European Medicine Agency (EMA), 2017).

Descriptive statistics were used to summarize data. Categorical data were reported as frequencies and percentages and compared using the Chi-square test, whereas continuous data were reported as median values with the related interquartile ranges (IQRs) and compared using the Mann-Whitney test. Logistic regression analyses were used to estimate the reporting odds ratios (RORs) with 95% confidence intervals (CIs) of ADE-related hospitalization, considering the following covariates: age, sex, ethnicity, number of implicated medications, parenteral administration, presence of interaction, therapeutic error, and/or complementary and alternative medicines (CAM).

Adjustment was performed for all the above mentioned covariates. All results were considered to be statistically significant at p < 0.05. Data management and statistical analysis were carried out using STATA 14.

The coordinating centre of Tuscany Region (Italy) approved MEREAFaPS Study (Notification number 1225 - December 21, 2009), and the local institutional ethics committee approved MEREAFaPS Study (Study number 3055/2010, Protocol number 45288 - August 6, 2014) according to the legal requirements concerning observational studies. Due to the retrospective nature of the present study and data anonymization, patient’s consent to participate was not required.

## Results

During the 12-year study period, a total of 61,855 ADE reports related to ED visits was evaluated; of them, 18,918 (30.6%) resulted in hospitalization. We estimated a rate of ADE-related visits and hospitalization of 5,154.6 and 1,576.5 reports per year, respectively. Considering that during the study period our monitors evaluated around 45millions of ED clinical charts, we estimated an overall incidence rate of 1.4 per 1,000 ADE-related ED visits and 0.4 per 1,000 ADE-related hospitalizations. **Table 1** and **Supplementary Table 1** report demographic and clinical characteristics of cases. Out of 61,855 ADE reports, 30,343 (49.0%) were defined as serious, and 160 (0.3%) of them were fatal. Most of ADEs occurred in females (n=35,010; 56.6%) and in Caucasians (n=54,232; 87.7%), with a mean (± standard error) patients’ age of 57.5 ± 25.0 years. A total of 31,460 (50.9%) ADEs had an improvement and 19,197 (31.0%) a complete resolution. For 8408 (13.6%) ADEs data on the outcome were not available. Overall, at time of ADE occurrence, patients were treated with more than 2 suspected drugs (n=35,853; 58.0%), and 49.2% of patients exposed to polypharmacy (≥5 concomitant drugs) were hospitalized. Visits were related to vaccines in 13.2% of cases; while CAMs were reported in 1.2% of reports. 1,574 (2.5%) cases of ADE were associated to drug abuse/misuse, and 1,436 (2.3%) were judged as preventable. Of them, 47% led to hospitalization. Dechallenge and rechallenge were positive in 49.6% and 0.8% of cases, respectively (data not shown). Among visits and hospitalizations, a statistically significant difference was observed for age groups (p=0.001), ethnicity (p < 0.001), number of suspected drugs (p < 0.001), type of medication (p < 0.001), presence of a suspected drug with parenteral administration (p < 0.001), type of event (p < 0.001), and preventability (p < 0.001).

**Table 1:** Case characteristics.

Case characteristic	ED visits for ADEs	ED visits for ADEsresulting in hospitalization	
	No. of cases 61,855 (%)	No. of cases 18,918 (row %)	
**Patient age, years**			
≤5	3,211 (5.2)	371 (11.6)	<0.0001
6–19	2,801 (4.5)	509 (18.2)	
20–64	26,039 (42.1)	6,469 (24.8)	
65–79	16,066 (26.0)	5,479 (34.1)	
≥80	13,175 (21.3)	5,817 (44.2)	
*Not available*	563 (0.9)	273 (48.5)	
Mean ± standard error, years	57.5±25.0	64.9±22.1	
**Sex**			
Female	35,010 (56.6)	10,592 (30.3)	0.042
Male	26,845 (43.4)	8,326 (31.0)	
**Patient ethnicity**			
Asian	892 (1.4)	186 (20.9)	<0.0001
Black or African American	550 (0.9)	154 (28.0)	
Caucasian	54,232 (87.7)	16,743 (30.9)	
Other	156 (0.3)	38 (24.4)	
*Not available*	6,025 (9.7)	1,797 (29.8)	
**No. of suspected drugs involved in the ADE**			
1	26,002 (42.0)	4,974 (19.1)	<0.0001
2	10,512 (17.0)	2,765 (26.3)	
3–4	10,397 (16.8)	3,828 (36.8)	
≥5	14,944 (24.2)	7,351 (49.2)	
**Type of medication**			
Drug	60,684 (98.1)	18,793 (31.0)	<0.0001
Vaccine	1,171 (1.9)	125 (10.7)	
**Presence of a suspected drug with parenteral administration**			
No	53,665 (86.8)	16,270 (30.3)	<0.0001
Yes	8,190 (13.2)	2,648 (32.3)	
**Presence of CAM**			
Yes	728 (1.2)	231 (31.7)	0.500
No	61,127 (98.8)	18,687 (30.6)	
**Type of event**			
Abuse/misuse	1,574 (2.5)	905 (57.5)	<0.0001
Interactions	539 (0.9)	271 (50.3)	
Overdose	387 (0.6)	166 (42.9)	
Therapeutic errors	832 (1.4)	352 (42.3)	
**Preventability**			
Yes	1,436 (2.3)	675 (47.0)	<0.0001
No	12,535 (20.3)	2,831 (22.6)	
Non assessable	5,207 (8.4)	1,695 (32.6)	
*Not applicable*	42,677 (69.0)	13,717 (32.1)	
**Total**	**61,855 (100)**	**18,918 (30.6)**	

**Table 2** reports the suspected drug classes. Out of 78,361 suspected agents, 16,348 belonged to the ATC class B (Blood and blood forming organs), 15,920 to the ATC class N (Nervous system), 14,664 to the ATC class J (Antinfectives for systemic use), 8,861 to the ATC class C (Cardiovascular system), 8,150 to the ATC class A (Alimentary tract and metabolism), 7,717 to the ATC class M (Musculoskeletal system), 1,722 to the ATC class L (Antineoplastic and immunomodulating agents), 1,459 to the ATC class R (Respiratory system), 1,146 to the ATC class H (Hormonal preparations), and 970 to the ATC class G (Genitourinary system and sex hormones).

**Table 2:** Suspected drug classes.

Drug class	ED visits for ADEs	ED visits for ADEs resulting in hospitalization
	No. of suspected agents 78,361 (%)	No. of suspected agents 26,335 (row %)
**Blood and blood forming organs**	16,348	6,156
Anticoagulants	10,252 (13.1)	3,899 (38.0)
Vitamin K antagonists (warfarin)	8,045 (10.3)	2,924 (36.3)
Factor Xa inhibitors	1,017 (1.3)	458 (45.0)
Unfractionated and Low-molecular-weight heparins	744 (0.9)	303 (40.7)
Direct thrombin inhibitors	446 (0.6)	214 (48.0)
Antiplatelets	5,802 (7.4)	2,180 (37.6)
Acetylsalicylic acid	4,153 (5.3)	1,523 (36.7)
Platelet P2Y_12_ receptor antagonists	1,508 (1.9)	603 (40.0)
Enzymes (alteplase)	141 (0.2)	54 (38.3)
Antihemorrhagic, antianemic and perfusion preparations	294 (1.1)	77 (26.2)
**Nervous system**	**15,920**	**6,426**
Analgesics	5,474 (7.0)	1,400 (25.6)
Opioid analgesics (codeine combinations)	2,988 (3.8)	783 (26.2)
Non-opioid analgesics (paracetamol)	2,333 (3.0)	586 (25.1)
Antimigraine preparations	153 (0.2)	31 (20.3)
Sedative or hypnotic agents	3,802 (4.8)	2,028 (53.3)
Benzodiazepines	2,659 (3.4)	1,437 (54.0)
Nonbenzodiazepine or nonbarbiturate sedatives	1,143 (1.5)	591 (51.7)
Antidepressants	2,140 (2.7)	971 (45.4)
Antipsychotics	1,967 (2.5)	933 (47.4)
Antiepileptics	1,814 (2.3)	862 (47.5)
Other nervous system agents	723 (0.9)	232 (32.1)
**Antinfectives for systemic use**	**14,664**	**2,660**
Antibacterials	12,389 (15.8)	2,350 (19.0)
Penicillins (amoxicillin-clavulanate)	7,057 (9.0)	1,190 (16.9)
Quinolones (levofloxacin)	1,975 (2.5)	485 (24.5)
Cephalosporins (ceftriaxone)	1,354 (1.7)	307 (22.7)
Macrolides (clarithromycin)	1,310 (1.7)	218 (16.6)
Sulfamethoxazole and trimethoprim	353 (0.4)	82 (23.2)
Other antibacterials	340 (0.4)	68 (20.0)
Vaccines	1,720 (2.2)	184 (10.7)
Antivirals and antiretrovirals	330 (0.4)	66 (20.0)
Other antinfectives agents	225 (0.3)	60 (26.7)
**Cardiovascular system**	**8,861**	**3,623**
Renin-angiotensin system inhibitors	2,880 (3.7)	1,116 (38.7)
Diuretics	1,575 (2.0)	897 (56.9)
Beta blocking agents	1,387 (1.8)	552 (39.8)
Calcium channel blockers	799 (1.8)	217 (27.1)
Antiarrhythmics	494 (0.6)	233 (47.2)
Lipid modifying agents	340 (0.4)	126 (37.0)
Digitalis glycosides (digoxin)	333 (0.4)	238 (71.5)
Antiadrenergic agents (doxazosin)	300 (0.4)	77 (25.7)
Other cardiovascular agents	753 (1.0)	167 (22.2)
**Alimentary tract and metabolism**	**8,150**	**3,748**
Diabetes agents	6,042 (7.7)	3,215 (53.2)
Insulin	3,654 (4.7)	1,819 (49.8)
Oral diabetes agents	2,388 (3.0)	1,396 (58.4)
Anti-ulcer and antacid agents	743 (0.9)	182 (24.5)
Propulsives (metoclopramide)	307 (0.4)	83 (27.0)
Antidiarrheals	283 (0.4)	65 (23.0)
Drugs for constipation	158 (0.2)	44 (27.8)
Stomatological preparations	152 (0.2)	30 (19.7)
Other gastrointestinal agents	465 (0.7)	129 (27.7)
**Musculoskeletal system**	**7,717**	**1,892**
Nonsteroidal anti-inflammatory drugs	6,617 (8.4)	1,616 (24.4)
Ketoprofen	1,876 (2.4)	418 (22.3)
Ibuprofen	1,607 (2.0)	333 (20.7)
Diclofenac	1,075 (1.4)	315 (29.3)
Nimesulide	570 (0.7)	162 (28.4)
Ketorolac	289 (0.4)	98 (33.9)
Naproxen	279 (0.4)	79 (27.6)
Etoricoxib	226 (0.3)	59 (26.1)
Others	695 (0.9)	152 (21.9)
Muscle relaxants (thiocolchicoside)	422 (0.5)	91 (21.6)
Antigout preparations (allopurinol)	300 (0.4)	122 (40.7)
Topical products	263 (0.3)	32 (12.2)
Other musculoskeletal agents	120 (0.1)	31 (25.8)
**Antineoplastic and immunomodulating agents**	**1,722**	**833**
Antineoplastic agents	1,252 (1.6)	679 (54.2)
Immune modulators	370 (0.5)	124 (33.5)
Endocrine therapy	100 (0.1)	30 (30.0)
**Respiratory system**	**1,459**	**282**
Nasal, cough and cold preparations	813 (1.0)	146 (17.9)
Bronchodilators	421 (0.5)	84 (19.9)
Antihistamines for systemic use	225 (0.3)	52 (23.1)
**Hormonal preparations**	**1,146**	**326**
Corticosteroids for systemic use	864 (1.1)	259 (30.0)
Thyroid therapy	250 (0.3)	54 (21.6)
Other hormonal agents	32 (0.0)	13 (40.6)
**Genitourinary system and sex hormones**	**970**	**191**
Systemic and vaginal contraceptives	467 (0.6)	92 (19.7)
Drugs used in benign prostatic hypertrophy (tamsulosin)	378 (0.5)	85 (22.5)
Other gynaecological agents	125 (0.2)	14 (11.2)
**Other agents**	**1,404**	**198**
**Total agents**	**78,361 (100)**	**26,335 (33.6)**

**Table 3** reports the most commonly reported suspected drugs leading to hospitalization by patient age. Overall, warfarin was the most reported one, followed by acetylsalicylic acid, amoxicillin-clavulanate, short-acting insulin, and lorazepam. Among patients aged ≤19 years, amoxicillin-clavulanate was the most reported drug, followed by paracetamol, ibuprofen, ketoprofen, and hexavalent vaccine. In elderly, hospitalization was mainly related to warfarin, acetylsalicylic acid, short-acting insulin, metformin, and furosemide use.

**Table 3:** Most commonly reported suspected drug products by patient age.

Drug product	ED visits for ADEs resulting in hospitalization
**All patients** **(N = 18,918 hospitalized out of 61,855)**	**No. of Suspected Agents** **26,335 (%)**
Warfarin	2,611 (9.91)
Acetylsalicylic acid	1,523 (5.78)
Amoxicillin-clavulanate	898 (3.41)
Insulin glargine (short-acting)	602 (2.98)
Lorazepam	469 (1.78)
Metformin	466 (1.77)
Ketoprofen	418 (1.59)
Furosemide	401 (1.52)
Insulin lispro (rapid-acting)	368 (1.40)
Alprazolam	360 (1.37)
Clopidogrel	352 (1.34)
Ibuprofen	333 (1.26)
Levofloxacin	321 (1.22)
Diclofenac	315 (1.20)
Acenocoumarol	313 (1.19)
**Patients aged ≤19 y** **(N = 880 hospitalized out of 6,012)**	**No. of suspected agents** **1,162 (%)**
Amoxicillin-clavulanate	83 (7.14)
Paracetamol	63 (5.42)
Ibuprofen	56 (4.82)
Ketoprofen	48 (4.13)
Diphtheria-hemophilus influenzae B-pertussis-poliomyelitis-tetanus-hepatitis B	35 (3.01)
Pneumococcus, purified polysaccharides antigen conjugated	33 (2.84)
Lorazepam	32 (2.75)
Carbamazepine	23 (1.98)
Amoxicillin	22 (1.89)
Valproic acid	20 (1.72)
Ceftriaxone	19 (1.64)
Clarithromycin	19 (1.64)
Alprazolam	17 (1.46)
Clonazepam	15 (1.29)
Measles, combinations with mumps and rubella, live attenuated	14 (1.20)
Metoclopramide	14 (1.20)
**Patients aged ≥65 y** **(N = 11,296 hospitalized out of 29,241)**	**No. of Suspected Agents** **15,335 (%)**
Warfarin	2,033 (15.19)
Acetylsalicylic acid	1,286 (8.38)
Insulin glargine (short-acting)	419 (2.73)
Metformin	377 (2.46)
Furosemide	349 (2.27)
Clopidogrel	302 (1.97)
Acenocoumarol	268 (1.75)
Metformina and sulfonylureas	258 (1.68)
Amoxicillin-clavulanate	246 (1.60)
Insulin lispro (rapid-acting)	240 (1.56)
Ramipril	217 (1.41)
Digoxin	213 (1.39)
Dabigatran	205 (1.34)
Levofloxacin	192 (1.25)
Glimepiride	184 (1.20)

**Table 4** reports adverse event manifestation by most commonly reported suspected drug classes. Anticoagulants and antiplatelet agents (n=16,054), antibacterials (n=12,389), nonsteroidal anti-inflammatory drugs (NSAIDs) (n=6,617), insulins and oral diabetes agents (n=6,042), opioid and non-opioid analgesics (n=5,321), and sedative or hypnotic agents (n=3,802) were the most commonly suspected agents for ED visits and hospitalization. Haemorrhagic events were frequently associated to anticoagulants and antiplatelet agents (50.5% of visits), moderate to severe dermatologic reactions and gastrointestinal disturbances to antibacterials (42.7% and 10.1%, respectively) and to NSAIDs (27.9% and 14.4%, respectively), hypoglycaemic events to antidiabetic agents (59.4%), and neurologic events to analgesics (22.7%) and to sedative or hypnotic agents (33.0%).

**Table 4:** Adverse event manifestation by most commonly reported suspected drug classes.

Adverse event manifestation	ED visits for ADEs	ED visits for ADEs resulting in hospitalization
	No. of preferred terms N (%)	No. of preferred terms N (row %)
**Anticoagulants + Antiplatelets** **N = 16,054**	**21,230**	**9749**
Haemorrhage	10,719 (50.5)	4,286 (40.0)
Epistaxis	3,559 (16.76)	346 (9.7)
Gastrointestinal	3,083 (14.5)	2,058 (67.0)
Genitourinary	1,221 (5.7)	429 (35.1)
Central nervous system	1,142 (5.4)	924 (80.9)
Dermatologic	1,018 (4.8)	407 (40.0)
Pulmonary	365 (1.7)	103 (28.2)
Ophthalmic	331 (1.5)	19 (5.7)
Altered international normalized ratio	1,966 (9.3)	524 (26.6)
Anaemia	994 (4.7)	880 (88.5)
Unintentional or intentional overdose	280 (1.3)	129 (46.1)
**Antibacterials** **N = 12,389**	**22,720**	**4,827**
Dermatologic reaction	9,710 (42.7)	1,546 (15.9)
Urticaria	3,208 (14.1)	441 (13.7)
Localized or general pruritus	2,674 (11.8)	442 (16.5)
Erythema	2,001 (8.8)	399 (19.9)
Rash	1,827 (8.0)	264 (14.4)
Localized or peripheral edema	1,484 (6.5)	324 (21.8)
Gastrointestinal disturbance	2,296 (10.1)	460 (20.0)
Nausea or vomiting	1,064 (4.7)	218 (20.5)
Abdominal pain	644 (2.8)	126 (19.6)
Diarrhoea	588 (2.6)	116 (19.7)
Unspecified hypersensitivity	1,050 (4.6)	203 (19.3)
Neurological effect	1,029 (4.5)	229 (22.2)
Respiratory reaction	844 (3.7)	233 (27.6)
Dyspnea	684 (3.0)	193 (28.2)
Throat tightness	160 (0.7)	40 (25.0)
Anaphylaxis	163 (0.7)	88 (54.0)
**Nonsteroidal anti-inflammatory drugs** **N = 6,617**	**12,157**	**3,269**
Dermatologic reaction	3,391 (27.9)	521 (15.4)
Urticaria	1,348 (11.1)	188 (13.9)
Localized or general pruritus	961 (7.9)	156 (16.2)
Erythema	552 (4.5)	97 (17.6)
Rash	530 (4.3)	80 (15.1)
Gastrointestinal disturbance	1,757 (14.4)	643 (36.6)
Abdominal pain	813 (6.7)	228 (28.0)
Nausea or vomiting	500 (4.1)	147 (29.4)
Melena	181 (1.5)	136 (75.1)
Gastritis	140 (1.1)	38 (27.1)
Hematemesis	123 (1.0)	94 (76.4)
Localized or peripheral edema	1,366 (11.2)	263 (19.2)
Unspecified hypersensitivity	434 (3.6)	73 (16.8)
Respiratory reaction	324 (2.7)	93 (28.7)
Abuse or self-harm	157 (1.3)	97 (61.8)
**Insulin + Oral diabetes agents** **N = 6,042**	**6,723**	**3,697**
Hypoglycaemia (from mild to severe)	3,006 (44.7)	1,560 (51.9)
Hypoglycaemia-related symptoms	992 (14.7)	634 (63.9)
Shock, loss of consciousness, or seizures	514 (7.6)	347 (67.5)
Altered mental status	191 (2.8)	124 (64.9)
Presyncope or syncope	152 (2.3)	74 (48.7)
Acidosis	135 (2.0)	89 (65.9)
Neurological effect	708 (10.5)	403 (56.9)
Drowsiness	231 (3.4)	154 (66.7)
Hyperhidrosis	173 (2.6)	90 (52.0)
Muscular weakness	170 (2.5)	84 (49.4)
Aphasia, dizziness, or tremor	134 (2.0)	75 (56.0)
Therapeutic error	261 (3.9)	136 (52.1)
Gastrointestinal disturbance	192 (2.8)	95 (49.5)
**Opioid + Non-opioid analgesics** **N = 5,321**	**11,237**	**3,001**
Neurological effect	2,549 (22.7)	568 (22.3)
Muscular weakness	615 (5.5)	111 (18.0)
Dizziness	558 (5.0)	81 (14.5)
Presyncope or syncope	355 (3.2)	92 (25.9)
Drowsiness	224 (2.0)	137 (61.2)
Hyperhidrosis	199 (1.8)	44 (22.1)
Altered mental status	199 (1.8)	77 (38.7)
Headache	139 (1.2)	26 (18.7)
Gastrointestinal disturbance	2,437 (21.7)	593 (24.3)
Nausea or vomiting	1,730 (15.4)	377 (21.8)
Abdominal pain	707 (6.3)	193 (27.3)
Constipation	78 (0.7)	26 (33.3)
Dermatologic reaction	1,587 (14.1)	229 (14.4)
Urticaria	592 (5.3)	72 (12.2)
Localized or general pruritus	479 (4.3)	75 (15.6)
Erythema	300 (2.7)	49 (16.3)
Rash	216 (1.9)	33 (15.3)
Localized or peripheral edema	287 (2.5)	60 (20.9)
Abuse or self-harm	190 (1.7)	111 (58.4)
Respiratory distress	189 (1.7)	52 (27.5)
Unspecified hypersensitivity	154 (1.4)	19 (12.3)
**Sedative or hypnotic agents** **N = 3,802**	**7,744**	**4,271**
Neurological effect	2,553 (33.0)	1,607 (62.9)
Drowsiness	1,595 (20.6)	994 (62.3)
Altered mental status or bradyphrenia	501 (6.5)	277 (55.3)
Loss of consciousness	158 (2.0)	110 (69.6)
Bradykinesia	157 (2.0)	112 (71.3)
Muscular weakness	142 (1.8)	48 (33.8)
Presyncope or syncope	130 (1.7)	66 (50.8)
Abuse or self-harm	1,962 (25.3)	1,273 (64.9)

**Figure 1:**
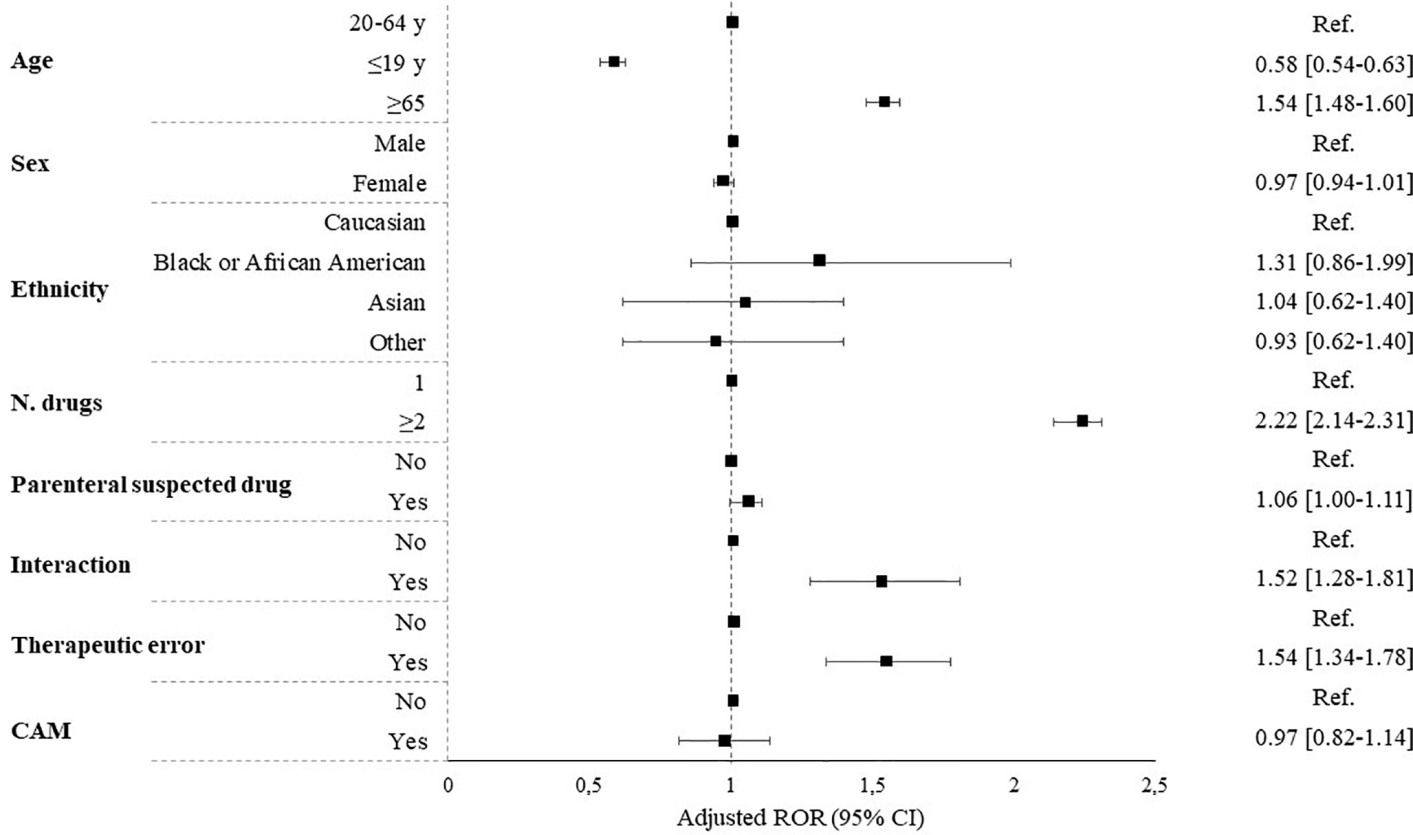
Predictors of hospitalization expressed as reporting odds ratio (ROR). CAM, complementary and alternative medicine.

Multivariate logistic regression analysis showed that, after adjustment for potential confounding, the risk of hospitalization was significantly higher for older age (ROR 1.54 [95% CI, 1.48–1.60]), higher number of concomitantly suspected drugs (ROR 2.22 [95% CI, 2.14–2.31]), presence of drug-drug interactions (ROR 1.52 [95% CI,1.28–1.81]), and therapeutic error (ROR 1.54 [95% CI, 1.34–1.78]) (**Figure 1**).

## Discussion

This study aimed to describe the frequency and the clinical characteristics of ADEs leading to ED visit and hospitalization in outpatients in Italy. As far as we know, this is the first nationwide pharmacovigilance study, conducted with an “active” approach, designed to estimate the risk of hospitalization associated with ADE, for a long period of observation and in a representative number of Italian EDs. For the first time, the characteristics of the entire MEREAFaPS Study database (demographic and clinical characteristics of patients, ATC classes, SOC, and PT) have been described. Based on the evidence herein reported, Italian medical doctors should be aware of the population subset and pharmacological treatments most frequently associated to ADEs in our country, considering that the elderly, females, exposure to more than two concomitant medications, and being treated with anticoagulants, analgesics, sedative or hypnotic agents and antibacterials are risk factors for ADE-related hospitalizations.

In the last decades, several studies have been published on ADEs as cause of ED visits and hospitalizations in European countries. Baena et al. (2014) performed a cross-sectional study in EDs of nine Spanish hospitals during a 3-month period. The overall prevalence of negative clinical outcomes of medications (NCOMs) was 35.7%. Authors estimated that about 81% of the NCOMs could have been prevented. Authors’ evidence confirmed their previous analysis in this field (Baena et al., 2006). In their second study, weighted prevalence of lack of safety, which could be compared with our incidence rate, was 2.4% (95% CI, 1.9–2.8). This difference is determined by another approach to data collection. In fact, authors used a questionnaire-based method to gather information about patients’ medications and related health problems.

Nickel and colleagues (Nickel et al., 2013) identified the frequency of drug-related problems (DRPs) among elderly patients presenting to the ED of the University Hospital Basel, Switzerland (May 2007-April 2009). Out of 633 patients, with a median age of 81 years and a mean Charlson comorbidity index of 2.5, 77 presented a DRP. 64 of them fulfilled the criteria “serious condition”. Polypharmacy and certain medications, in particular thiazides, antidepressants, benzodiazepines, anticonvulsants, were the most frequently causes of DRPs. Focusing on the elderly patients in our sample, we observed different pharmacological classes than described by Nickel and colleagues. Particularly, anticoagulants and antiplatelet agents, antidiabetic medications (including insulin), and furosemide were the most frequently reported. Notably, these medications are characterised by a high prevalence of use in Italy (National Observatory on the Use of Medicines (OsMed), 2019), but, differently from medications reported by Nickel (i.e., thiazides, benzodiazepines, etc.), they are generally not inappropriate in this subset.

Roulet et al. (2013) estimated the frequency and the severity of drug-related visits in ED, in order to assess ADE recognition by emergency physicians (October 2007-31 March 2008). In their prospective cross-sectional single centre study in France, a total of 95 out of 423 eligible patients experienced an ADE. Emergency physicians correctly attributed 33 of these cases to a medication-related problem. Authors concluded that ADEs are frequent in EDs and are not always well recognised by emergency physicians, especially when the drug is involved in a multifactorial pathological condition. Comparing this limitation to our methodology, since our monitors evaluated all ED clinical charts with a positive anamnesis for the presence of a pharmacological treatment, we could consider that the involvement of trained monitors may allowed us to recognise and collect the majority of ADE-related ED visits.

In Finland, Juntti-Patinen and colleagues (Juntti-Patinen et al., 2006) determined the incidence of drug-related ED visits to a district hospital, and identified the drugs and patient groups involved over a period of 6 months. Of the 7,113 evaluated visits, 2.3% were “certainly” or “probably” drug-related, 1.4% were related to adverse drug reactions (ADRs) and 0.9% to intentional overdoses. The most common ADRs were gastrointestinal symptoms caused by antibiotics, opioids, NSAIDs, or cytostatic drugs. The ADR patients were older than the intentional overdose patients. Males predominated in the intentional overdose group but not in the ADR patients. Notably, in our sample we observed different kind of ADEs, in particular haemorrhages for anticoagulant and antiplatelet agents, and dermatological reactions for both antibacterials for systemic use and NSAIDs. These differences are probably driven by the demographic and clinical characteristics of the Italian and Finnish populations.

In Italy, Rosafio and colleagues (Rosafio et al., 2017) retrospectively analysed data for children seeking medical evaluation for a medication-related visits over an 8-year period in a single tertiary centre. They found a total of 497 medication-related visits, 54% of which occurred in children from 0 to 2 years of age. The most common events were related to ADRs (30.3%). The medication classes most frequently implicated in an ADE were anti-infective drugs for systemic use, central nervous system agents and respiratory system drugs. The most common symptom manifestations were dermatologic conditions, general disorder and administration site conditions and gastrointestinal symptoms. Another single-centre study performed in Italy on children (Lombardi et al., 2018) confirmed the evidence reported by Rosafio. In addition, young age and polypharmacy were found to be predictive of ADR seriousness. Capuano et al. (2009) described the characteristics of ADEs in 10 EDs of general hospitals in southern Italy. Comparable to our evidence, authors reported that ADEs were significantly more frequent in women, in elderly, and in patients exposed to antibiotics, NSAIDs, and agents acting on the renin-angiotensin system. Also in this case, authors’ evidence confirmed their previous results published in 2004 (Capuano et al., 2004). Trifirò and colleagues (Trifiro et al., 2005), during the year 2000, performed a prospective study in two observational periods of 10 days each in 22 Italian EDs. On 18,854 enrolled patients, 629 (3.3%) were affected by ADE. Among these, around 39% of ADE patients reported a serious event. Patients with ADE were significantly more likely to be hospitalized, females and elderly, compared with the total sample. NSAIDs and antibiotics were the drugs mostly involved in ADE occurrence, which affected mostly the cutaneous and gastrointestinal systems. Although the above mentioned evidence is comparable to that observed in our study, particularly for gender, age groups, drug classes, ADE types, seriousness and preventability, in Europe most of the studies were performed in a single centre, with a short period of observation, therefore considering a small sample of patients and fewer adverse events. Actually, only an Italian evaluation has already been published on data obtained by the MEREAFaPS initiative (Perrone et al., 2014). This article was the first published in Italy, however it reported data from the EDs enrolled in only one Region during a short observational period. Actually, our data include those already presented by Perrone et al., who described the impact of ADRs through a retrospective 2-year analysis performed in 32 EDs of Lombardy region (northern Italy). In their study authors collected a total of 8,862 ADRs with an overall prevalence rate of 3.5 per 1,000 visits. Of all ADRs, 42% were probably/definitely preventable and 46.4% were serious, 15% required hospitalization, and 1.5% resulted in death. The SOC most frequently associated with ADRs were: skin and subcutaneous tissue, gastrointestinal, respiratory thoracic and mediastinal, and nervous system disorders. The most common ATC classes involved in ED visits were anti-infectives and immunomodulating agents, blood and blood-forming organs agents, and nervous system agents. Older age and higher number of concomitantly taken drugs were significantly associated with an increased risk of hospitalization. Therefore, except for estimates regarding the incidence rate per 1,000 visits (1.4 versus 3.5), evidence described by Perrone is completely comparable to that we observed now at Italian national level.

Comparing our evidence with that obtained from different population setting, such as the American one, the most frequently suspected drug classes implicated in ED visits and hospitalizations for ADEs in Italy are the same identified by Shehab and colleagues in the United States (Shehab et al., 2016). In their study, anticoagulants, antibiotics, antidiabetics, analgesics, and cardiovascular agents were implicated in the majority ED visits and hospitalizations for ADEs, which included clinically significant ADEs, such as haemorrhage (anticoagulants), allergic reactions (antibiotics), hypoglycaemia (diabetes agents), and moderate to severe neurological effect (analgesics). Of notice, the differences observed for medications belonging to the ATC class N, in particular for opioid analgesics and sedative or hypnotic agents. In our sample, ATC class N is the second most frequently reported therapeutic group, while in the United States the nervous system agents represent the fourth cause of ED visits and hospitalization for ADE.

Interestingly, in Italy the frequency of ED visit and hospitalization seems to be higher for sedative or hypnotic agents once compared with other drug classes inducing both acute or chronic toxicity (i.e., substance use disorder), such as opioids. In fact, in our sample 53.3% of patients exposed to sedative or hypnotic agents and admitted to the ED were hospitalized, versus 25.6% of patients exposed to analgesics. Similarly, differences in hospitalization frequency can be observed for other medication groups. Further investigations should be performed for direct thrombin inhibitors (48% of hospitalizations), antidepressants (45%), antipsychotics (47%), antiepileptics (47%), diuretics (57%), and oral diabetes agents (58%).

The evidence provided in this article is comparable to the majority of international publications (Zed et al., 2013), in terms of patients’ characteristics, drug classes and ADEs most frequently reported. The highest percentage of female patients with ADE observed in our EDs can simply reflect the demographic data, since in Italy there are more female patients (Italian Statistics Bureau (ISTAT), 2018), especially those over the age of 65 years. Considering the characteristics of ADEs, they are mainly common and related to the pharmacokinetics and pharmacodynamics of the suspected drugs. Notably, our results highlighted that patients exposed to two or more concomitant suspected drugs were at statistically significant higher risk of hospitalization compared to those exposed to only one suspected drug. Therefore, our results may help identify which pharmacological classes need a special attention from professionals and healthcare systems in order to better recognise ADEs in outpatients.

In summary, the elderly, females, patients exposed to more than two concomitant medications, and those treated with anticoagulants, analgesics, sedative or hypnotic agents and antibacterials are at higher risk for a hospitalization related to an ADE in Italy. Taking into account all these features could help both general practitioners and ED physicians to prevent and better manage ADEs. This update and an improvement of appropriateness of prescription and use of medications, go hand in hand to ameliorate drug safety.

Although an individual patient data meta-analysis highlighted that risk factors associated with ADEs in hospitalized patients are quite comparable with those observed in our sample for out-patients, since we excluded these cases from our analysis, it could be interesting to perform future investigations on this topic, comparing them with evidence already published (Sakuma et al., 2012; Boeker et al., 2015; Sakuma et al., 2020).

### Strengths and Limitations

There are a number of study limitations. First, this study includes only ADEs diagnosed and treated in EDs. Furthermore, the evaluated setting, trained monitors’ expertise (i.e., clinical pharmacology and clinical pharmacy), and the methods used to identify ADEs can lead to a reporting bias that favoured the detection of events, which are generally more common and serious, therefore easier to detect (i.e., bleeding and hypoglycaemia). Nevertheless, having considered all cases that reported a pharmacological treatment in anamnesis may have reduced the reporting bias. Second, the retrospective nature of the study may have led to an underestimation of ADEs rate, since not all patients presenting an ADE, even if serious, attend ED or spontaneously report the ADE. However, considering that data on ADE reports were collected through a national active pharmacovigilance initiative, the issue of underreporting can be considered of relatively low relevance. Third, our analysis is based on ADE reports that are affected by limits that include inaccurate and incomplete information, mainly related to lack of clinical data also in the ED electronic sources (i.e., clinical chart and hospital discharge database). Given that, the absence of information that was not listed in ADE reports and that might have influenced the clinical evaluation of each report (i.e., the lack of information on previous and/or current patient medical conditions which could affect the clinical evaluation of each case) could not always be excluded, particularly for the assessment of preventability. Despite these limitations, this is the first nationwide multicentre study performed in Italy on the ADEs associated to ED visits and hospitalizations, over 12-year of observation. Moreover, our study involved different catchment areas of Italy (hospitals are equally distributed through the national territory), thus the evidence herein reported can be considered representative of all Italian EDs.

## Conclusion

Targeting ADEs common among specific patient populations, such as the elderly (age ≥65 years), women and patients exposed to two or more concomitant suspected drugs, may help further focus on outpatients’ medication safety in order to early recognise and prevent ADEs. In the frame of EDs, active pharmacovigilance studies represent the best observational methodology, allowing healthcare professionals and systems to detect, collect and characterise the clinical burden of ADEs in outpatients. The MEREAFaPS Study described here is the largest conducted on ADE reports in Europe and can represent a reliable data source to carry out real-world drug-safety studies. Furthermore, to put our data and identified risk factors associated with hospitalization due to ADEs into a future perspective, it will be important to compare our sample with a large group of ED patients without ADEs, hospitalized, and not hospitalized. Further analysis is certainly need and will be carried out in the future.

## Data Availability Statement

The datasets generated for this study are available on request to the corresponding author.

## Author Contributions

Study design was contributed by NL, GC, AB, and AV, with assistance from the rest of the authors. AB took the lead in data analysis, assisted by RB, NL, and GC. Data interpretation was performed by NL, GC, and AV, with assistance from the other authors. The manuscript was written primarily by NL and GC, with assistance from the other authors, and revised by MT, AC, AM, MV, and GV. All authors approved the final version of the manuscript.

## Funding

This study was funded by a research grant from the AIFA (the 1091 Italian Medicines Agency), Rome, Italy. The funder of the study had 1093 no role in the collection, analysis and interpretation of data, nor 1094 in the writing of the report, nor in the decision to submit the 1095 article for publication.

## MEREAFaPS Study Group

Members of the MEREAFaPS Study group who provided patient data for this study: Maria Luisa Aiezza (Naples), Alessandra Bettiol (Florence), Daria Bettoni (Brescia), Corrado Blandizzi (Pisa), Roberto Bonaiuti (Florence), Valentina Borsi (Florence), Annalisa Capuano (Naples), Errica Cecchi (Prato), Irma Convertino (Pisa), Giada Crescioli (Florence), Martina Del Lungo (Florence), Cristina Di Mauro (Naples), Gabriella Farina (Milan), Sara Ferraro (Pisa), Annamaria Fucile (Naples), Elena Galfrascoli (Milan), Elisabetta Geninatti (Turin), Linda Giovannetti (Florence), Luca Leonardi (Pisa), Rosa Liccardo (Naples), Niccolò Lombardi (Florence), Anna Marra (Ferrara), Eleonora Marrazzo (Turin), Giovanna Monina (Gallarate), Alessandro Mugelli (Florence), Silvia Pagani (Vimercate), Maria Parrilli (Florence), Concetta Rafaniello (Naples), Francesco Rossi (Naples), Marco Rossi (Siena), Stefania Rostan (Naples), Marco Ruocco (Vimercate), Marita Sironi (Vimercate), Giulia Spada (Vimercate), Liberata Sportiello (Naples), Marco Tuccori (Pisa), Alfredo Vannacci (Florence), Mauro Venegoni (Vimercate), Giuditta Violetta Vighi (Vimercate), Giuseppe Danilo Vighi (Vimercate).

## Conflict of Interest

The authors declare that the research was conducted in the absence of any commercial or financial relationships that could be construed as a potential conflict of interest.
