# Supplementary material for: Italian Emergency Department Visits and Hospitalizations for Outpatients’ Adverse Drug Events: 12-Year Active Pharmacovigilance Surveillance (The MEREAFaPS Study)
Source: Front Pharmacol. 2020 Apr 6;11:412. doi: 10.3389/fphar.2020.00412 (PMC7153477; doi:10.3389/fphar.2020.00412)
Supplement: Supplementary file 1 [file Table_1.docx]

| **Seriousness** |  |
| --- | --- |
| **Non-serious** | 31,512 (50.9) |
| **Serious** |  |
| Hospitalization or prolonged hospitalization | 18,918 (30.6) |
| Other clinically relevant condition | 9539 (15.4) |
| Life threatening | 661 (1.1) |
| Death | 160 (0.3) |
| Severe or permanent disability | 19 (0.03) |
| Congenital abnormality | 5 (0.0) |
| *Not available* | 1041 (1.7) |
| **Outcome** |  |
| Improvement | 31,460 (50.9) |
| Complete resolution | 19,197 (31.0) |
| Still unresolved | 2314 (3.7) |
| Death | 281 (0.5) |
| Resolution with sequelae | 195 (0.3) |
| *Not available* | 8408 (13.6) |
| **Dechallenge (N = 45,630)** |  |
| Positive | 22,632 (49.6) |
| **Rechallenge (N = 30,092)** |  |
| Positive | 244 (0.8) |

**Supplementary Table 1 -** Case characteristics by seriousness and outcome.
